# Are respectful maternity care (RMC) interventions effective in reducing intrapartum mistreatment against adolescents? A systematic review

**DOI:** 10.3389/fgwh.2023.1048441

**Published:** 2023-03-01

**Authors:** Helen H. Habib, Jefferson Mwaisaka, Kwasi Torpey, Ernest Tei Maya, Augustine Ankomah

**Affiliations:** Department of Population, Family and Reproductive Health, School of Public Health, College of Health Sciences, University of Ghana, Accra, Ghana

**Keywords:** respectful maternity care, adolescent sexual reproductive health and rights, intrapartum mistreatment, adolescent pregnancy and childbirth, patient – centered care

## Abstract

Intrapartum mistreatment of women by health professionals is a widespread global public health challenge. It leads to a decreased quality of maternity care and is evinced to precipitate detrimental maternal and neonatal outcomes, especially among adolescents. Relatedly, research indicates that Respectful Maternity Care (RMC) interventions are especially effective in mitigating intrapartum mistreatment and improving birth outcomes. However, evidence on the success of RMC, specifically for adolescents, is insufficient and unaggregated. Accordingly, this review specifically aims to synthesize existing evidence on RMC care provision to adolescent parturients. This review searched for relevant literature from published and gray sources including PubMed, ScienceDirect, Cochrane, CINAHL, PsycINFO, Scopus, as well as Population Council, WHO and White Ribbon Alliance data sources published between January 1990 and December 2021. Based on eligibility, studies were selected and quality appraised after which thematic analysis and narrative synthesis was conducted. Twenty-nine studies were included in the systematic review. Due to paucity and heterogeneity of quantitative studies, the review was limited to a thematic analysis. Adolescent and health provider perspectives alike underscored the burden and outcomes of mistreatment. Need for RMC interventions to improve quality of maternity care was recommended by majority of studies.

**Systematic Review Registration:**

https://www.crd.york.ac.uk/prospero/, identifier: CRD42020183440.

## Introduction

Globally, intrapartum mistreatment encountered by women during facility-based childbirth is documented as a detrimental, yet pervasive experience encountered by women in all socio-political contexts (1, 2). Intrapartum mistreatment, also referred to sometimes as disrespect and abuse, is widely acknowledged doubly as a human rights and public health challenge (3). This is because it not only infringes on women's rights to the best quality care but is also strongly associated with detrimental maternal and neonatal health outcomes including prolonged labour, injuries and death (4). Considering these consequences of intrapartum mistreatment, research and programmes have been dedicated towards investigating effective approaches to mitigate this grave situation. Facility-based births, assisted by skilled birth attendants is a key strategy which facilitates early identification and timely management of any complications that may arise during labour (5). Relatedly, reported statistics on facility-based births in sub-Saharan Africa (SSA) have largely demonstrated increments over the past decade (6). However, utilization of facility-based childbirths remains less than universal. This has been mainly attributed to the reluctance of a significant proportion of women to deliver babies in health facilities due to experiences of mistreatment and a general lack of respectful maternity care (7, 8). Indeed, even in contexts where rates of facility-based births are moderately high, women continued to expressed discontent with labour experiences and outcomes, mostly due to encounters of mistreatment (9).

Following widespread reports of intrapartum mistreatment, the WHO Human Reproductive Program (WHO-HRP) proposed recommendations to improve the quality of care of maternal health service delivery with respectful maternity care as an essential component (10). The WHO describes Respectful Maternity Care (RMC) as the organization and management of health systems in a manner that prioritizes respect for women's sexual and reproductive health and human rights (11). RMC, which is sometimes termed as compassionate care, denotes maternal healthcare that highlights positive interpersonal relations between healthcare providers and clients. This includes maintaining the dignity, confidentiality and privacy of clients, whilst ensuring continuous support and informed choices during childbirth. The theoretical underpinning of RMC is grounded in the fact that all women have the fundamental right to dignified and respectful care during childbirth and that intrapartum mistreatment doubles as a violation of women's sexual and reproductive health rights and also as a leading public health barrier. Despite RMC being a fundamental right of all women, research seems to suggest that there are certain vulnerable sub-groups of women, including younger, poorer, less educated, physically challenged, HIV-positive and ethnic minority women, who inordinately suffer a health inequity in receiving RMC (2, 12–14). Especially among adolescents, these correlates confer a higher risk of being denied RMC due to the intersectionality of their demographic characteristics; younger age, lower socio-economic class and lower level of education/literacy (15). This is further aggravated by provider-side factors including moral biases against adolescents engagement in early/pre-marital sex (9, 15). These issues are problematic for the health outcomes of adolescents as younger parturients are proven to bear an disproportionately elevated risk of poor maternal health outcomes than older parturients (16). In light of these challenges, adolescent parturients should ideally be supported with an emphasis on interpersonal experience (17, 18).

Globally, interventions have been developed to improve RMC delivery by healthcare professionals utilizing diverse strategies including sensitization and education of parturients about their rights to Sexual and Reproductive Health (SRH) and promoting legal channels for resolution of actionable cases (19, 20). Whilst these strategies have recorded promising results among older populations of women, these interventions appear to have limited success in meeting the unique needs and challenges of adolescents as a sub-population. Typical illustrations include the inability of adolescents to exercise their rights to sexual and reproductive health services due to some of their aforementioned characteristic vulnerabilities and their lack of access to the financial resources needed to pursue judicial remedy. This evidently creates a need to review the evidence on interventions that are designed with specific consideration for meeting the RMC needs of adolescents. The successes, challenges and lessons learnt from implanting these interventions must also be aggregated. This is to better inform and strengthen the design and implementation of future interventions aimed at providing quality RMC for adolescent parturients. The overall aim of this systematic review is to synthesize the existing evidence on respectful maternity care interventions specifically targeted at addressing adolescent intrapartum mistreatment.

The specific objectives of this systematic review are to

1. Review the existing evidence on the types and characteristics of RMC interventions that are specifically designed to meet the needs of parturient adolescents
2. Review the available evidence on the strategies, outcomes, gaps, challenges and lessons in the implementation of adolescent RMC interventions

## Methods

The protocol for this review has been registered in the International Prospective Register of Systematic Reviews (PROSPERO) under code CRD42020183440. This review is being reported according to the Preferred Reporting Items for Systematic Reviews and Meta-Analyses Protocols (PRISMA-*P*) statement and checklist (21).

### Search methods and identification of studies

Searches for research studies were conducted in electronic databases including PubMed, MEDLINE, Cochrane, ScienceDirect, CINAHL, Scopus, Web of Science, PsycINFO and Google Scholar. The reference lists from some relevant papers were also searched for related studies. Furthermore, gray literature searches of organizational websites of World Health Organization, White Ribbon Alliance, Population Council and USAID were conducted for any relevant information. Emails were sent to authors whose studies were not available in the public domain but were deemed relevant, to request private access to their articles. Searches were conducted using search strings of medical subject headings (MeSH) including the terms “Intrapartum Mistreatment”, “Disrespect and Abuse”, “Respectful Maternity Care”, “Person-Centered”, “Compassionate care”, “Adolescents”, “Teenager”, and “Pregnancy” in combination with the BOOLEAN operators (“AND”/“OR”). The search results were exported to Mendeley reference manager for cleaning and management.

### Eligibility criteria

The Population, Intervention, Comparator, Outcome and Study design (PICOS) framework guided the selection of studies. The studies were selected as follows:

**Participants:** Only studies that focused on adolescent parturients as the main study population or sub-analyses population of interest were deemed eligible. In the context of this study, parturient adolescent includes participants between ages 10 and 19 years who had had a childbirth.

**Interventions:** Interventional studies whose objectives mentioned interventions that provide respectful or compassionate care for adolescents were deemed eligible. Studies which represented adolescent perspectives and experiences of RMC and or quality of care interventions were also considered eligible.

**Comparators:** Comparator studies included research which compared facilities or programmes that provide the normal or standard quality of care package for parturients to facilities and programmes that are not specifically designed for RMC promotion.

**Outcome:** Studies that reported on the outcomes of interest i.e., experiences of RMC were included. Related outcomes that were considered eligible included reported satisfaction with quality of care and documented maternal and neonatal physical and psychosocial outcomes.

**Study design:** Study designs that were deemed eligible were primary quantitative cross-sectional, experimental, cohort (prospective and retrospective), interventional and case control studies. Qualitative observations and research of respectful care experiences were also deemed eligible. Studies published in English between January 1, 1990, and December 30, 2021, were eligible for inclusion. This timeline was selected to reflect the era from which the concept of respectful maternity care gained traction in the 1990s to the most recent studies of 2021.

### Study selection

**Figure 1:**
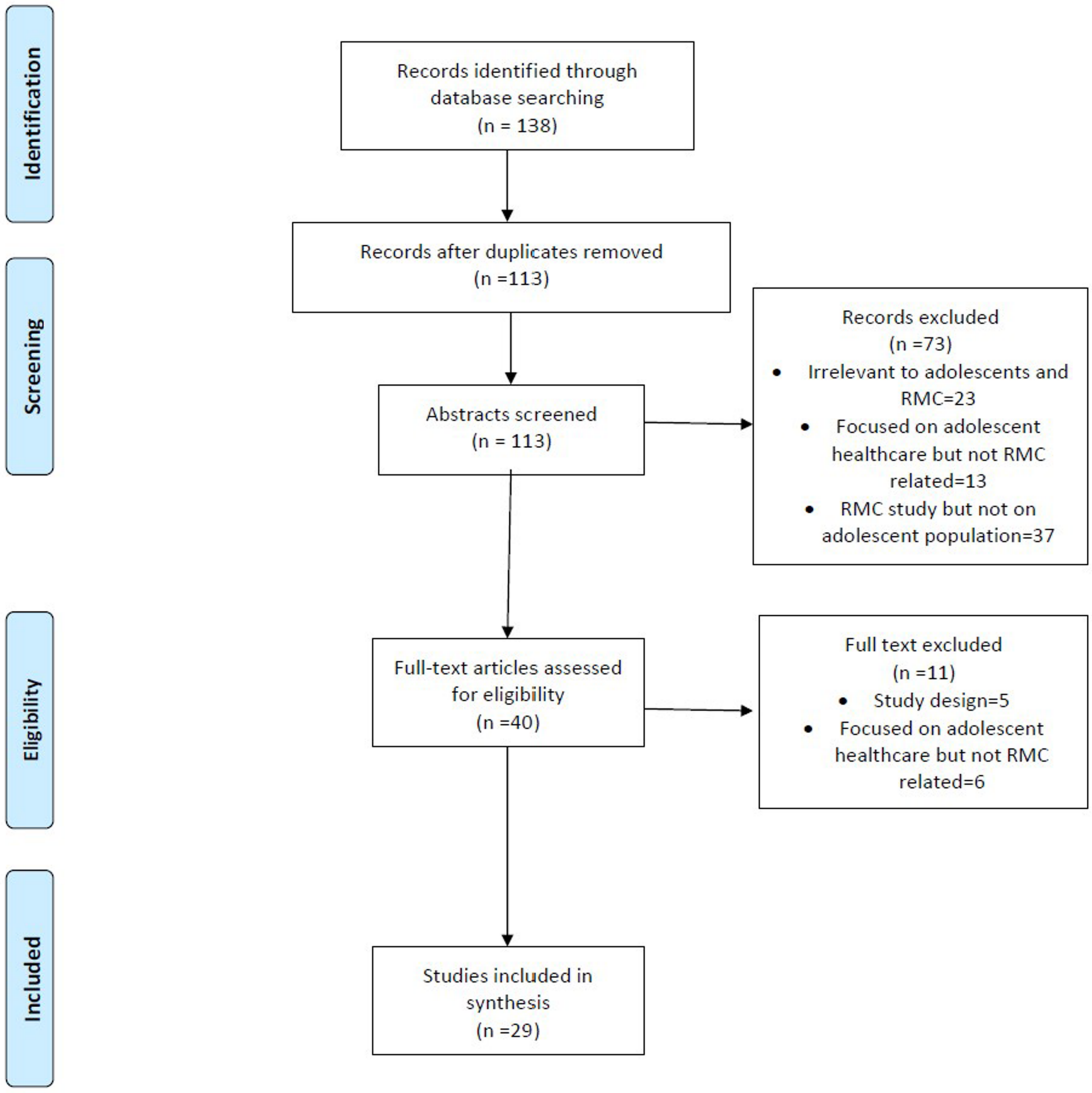
PRISMA Flowchart of study selection..

Using the selected search terms (Supplementary Material S1), 138 studies were identified from the databases. The identified studies contained some duplicates which led to 113 studies remaining after de-duplication. The studies from the searches were screened according to the eligibility criteria in three concurrent steps. Studies were initially screened according to their titles and abstracts which clearly reported on intrapartum mistreatment and RMC. A total of 113 abstracts were screened. Studies whose title and/or abstract screening were found to be irrelevant to both adolescents and the topic, were focused on adolescents healthcare but not (dis)respectful care or were focused on (dis)respectful care but did not contain an adolescent population were excluded at this stage. At this stage, 73 studies were removed. In the next stage of screening, the 40 full text versions of the studies that had passed through the prior eligibility steps were read and appraised for final inclusion into the systematic review. After the full text appraisal, 29 studies were included in the review as the remainder of studies did focus on adolescent healthcare but not (dis)respectful care The study selection process is represented by the PRISMA flowchart as shown in Figure 1. All the screening steps were conducted in duplicate by two reviewers, HHH and JM. In situations where the reviewers faced an impasse on studies, a third reviewer was involved to call the final decision. The outputs of the initial search results were transported into Mendeley reference manager to allow orderly download and storage of the selected studies and also to facilitate ease of shared access by all the involved reviewers.

### Data extraction and analyses

**Extraction**: The screening was conducted with the aid of a contextually developed MS-Excel extraction tool (Supplementary Material S2) With the aid of the data extraction tool, details of each study were extracted from full text manuscripts. These details included the author details, study design, study location (country), study population, sample size, aims, intervention and outcomes assessed. Information on key findings, gaps and recommendations was also extracted. Details of extracted information are presented in Supplementary Material S3. Extraction was conducted independently by HHH and JM. In the event of conflict in extractions between the two authors, a joint discussion was held, and a consensus agreed upon.

**Synthesis**: Following the extraction process, data was synthesized from the selected studies in a thematic analysis. This was performed by developing a thematic framework based on the themes extracted from the selected studies. The findings from the studies were then mapped to this framework. When novel themes were identified i.e., not already pre-occurring in the existing framework, these newly arising themes were used to update the existing framework. Similarities and contradictions between the findings of the studies were grouped and the themes were subjected to appraisal by the authors and agreed upon by consensus.

**Analyses**: the studies were screened for reported prevalence of intrapartum mistreatment and or respectful maternity care in order to explore the possibility of conducting a meta-analysis. However, majority of the studies selected did not report sufficient results on the prevalence, risk factors and other quantitative aspects of RMC to enable the conduct of a meta-analyses. This led to a conclusion to only conduct a qualitative systematic review of the studies.

### Quality assurance

Specific measures were taken to ensure the quality of this review as explained in the following section. Firstly, both published and grey literature sources were included in the review to mitigate against publication bias. Additionally, Preferred Reporting Items for Systematic Reviews and Meta-Analyses (PRISMA) guidelines were observed during the conduct of this review (21, Supplementary Material S4). Furthermore, the screening and review of the participating studies were conducted in duplicate by two independent reviewers, with the involvement of a third reviewer if there was an impasse between the two reviewers in screening or analyses. In addition, the methodological tools used in this review including eligibility, screening and data extraction tools were methodologically designed to suit the aim and objectives of the review. Also, studies underwent individual quality assessments; employing the relevant Joanna Briggs Institute critical appraisal tool suitable for each study design. Criteria which were assessed included congruity between the research aims and objectives and its philosophical underpinning. The relevance and suitability of the utilized methodology for the study design was also assessed.

## Results

Overall, 138 hits were identified from the database searches. Out of these, 25 duplicates were removed, leaving 113 manuscripts. Following abstract screening, 73 manuscripts were excluded due to reasons such as focusing on a topic that was deemed irrelevant to adolescents or focusing on adolescent health but not specifically RMC. Following this, 40 papers underwent full text screening. Another 11 publications were eliminated at this stage based on their study design and focusing on adolescent health but not directly relating to RMC. Ultimately, 29 studies were included in the final analyses. The steps taken through identification and screening are visually represented by the PRISMA diagram in Figure 1.

### Incidence and consequences of RMC

There was a paucity of studies that reported quantitatively on Respectful Maternity Care with only six quantitative studies being included in the study. Moreover, among these quantitative studies, substantial heterogeneity existed first in the definition of RMC, and furthermore in the reported prevalence. This created difficulties in accurately conducting a meta-analysis of the quantitative results. Nonetheless, the studies that conducted a prevalence analysis generally reported a decreased odds of receiving RMC correlating to certain factors such as younger age (22–25), higher parity (22), lower education (22, 24) and less wealth (22, 24). Studies in this review found the receipt of disrespectful care to often be correlated with complication or poor clinical outcomes (25). Some studies further made the correlation between the absence of RMC, a reduced incidence of facility-based deliveries and a consequent increase in maternal mortality rates (23). The consequence of a lack of respectful care also extended beyond the mothers but also to their children, as another study reported that following a negative childbirth experience, adolescent mothers were particularly mistrustful of the health system and often disengaged from seeking healthcare for their neonates, often leading to neonatal and other childhood complications (26). This raises serious public health concern as another vulnerable group is again affected by the lack of respectful care. Conversely, adolescent participants in the studies who felt treated with respect and a lack of judgement expressed confidence in being able to approach health providers with their own or their infants health concerns. A study in this review which sought the perspectives and introspection of health providers also proffered the viewpoint of a lack of professional satisfaction when adolescent mother were unhappy with the care they received, especially in relation to the interpersonal and emotional aspects of care (27).

### Adolescent perspectives of RMC

Seventeen studies focused entirely on adolescent perspectives of respectful maternity care; seeking to understand adolescent lived experiences and viewpoints of maternal care. Themes mostly portrayed adolescents describing mainly negative treatment by health professionals during labor and delivery. These studies underscore the focus of studies on experiences of intrapartum mistreatment with relatively little insight on strategies to curb this menace. This highlights the gap in literature on RMC interventions that are effective, especially for adolescents. From the studies, adolescents' perspectives usually focused on physical infrastructural challenges as well as poor health provider attitudes. In terms of health provider attitudes, studies often repeated themes such as “disrespectful, judgmental, humiliating, abusive and uncompassionate,” The concerns of adolescents from the studies were multi-faceted but were broadly centered around provider and infrastructural concerns. These concerns were described in studies with common cross-cutting themes such as “judgmental, disrespectful, humiliating, uncompassionate, abusive” (23, 26–28).

A few studies featured adolescents describing their desired form of care from professionals in frequently repeated terms of “caring, supportive, adolescent friendly, non-judgmental or unbiased, individualistic, dignified, and at ease” (26, 29–32). These terms succinctly described the kind of care idealized by the concept of Respectful Maternity Care. Most consistently, these recurring themes expressed the desire for a healthier interpersonal approach by providers. Studies also frequently described the specifically desired elements of care. A recurrently requested element of RMC was the permission of birth companionship which was repeatedly requested for in five studies (22, 29, 30, 33, 34); These studies all concluded in their results that adolescents perceived and actually encountered better maternal outcomes with known companions in attendance. Another area of RMC often described as important to adolescents was short waiting times whilst they received service (29, 35). According to most of the studies that investigated the perspectives of adolescents about quality of care during delivery, RMC was ranked highly, perhaps even the most important determinant of quality for the participants' care (27, 30, 36, 37). Only two studies paid direct attention to the contextual nuances of (dis)respectful maternity care and examined the ethnic contexts of care (38, 39).

### Health provider perspectives

Eleven studies altogether reported on provider perspectives of RMC (25, 27, 39–47). The provider perspectives featured broad themes firstly on the socio-political contexts for delivery of care, secondly on the awareness of RMC and the multi-faceted challenges associated with its delivery to adolescents and thirdly, the need for interventions, including infrastructural and behavioral interventions to promote RMC delivery. Studies which explored the socio-political dynamics of the relationships between providers and adolescents were mostly defined by the differences between the two groups often along the lines of age, educational level, marital status and socio-economic position. One of such studies highlighted these differences by exploring the cultural nuances which came into play between American nurses and adolescents in a small migrant community (39). Whilst health professional perspective studies oftentimes featured high self-ratings in the delivery of general obstetric care, there was also a recurrent revelation that providers doubted their own self-efficacy in providing RMC. Correspondingly, the health provider perspectives also suggested that enabling interventions such as behavioral skills training would be beneficial in improving RMC service delivery (18).

Provider perspectives correlated at some points with adolescents; with the allowance of birth companions as a component of RMC being one of such points of convergence. One study which highlighted the role of doulas (professional labor assistant who provide physical and emotional support to the parturient) as birth companions of adolescents (41). accentuated the benefits of birth companions to adolescents as they frequently have high emotional support needs which often are not met by obstetric providers (41). It was also argued that the allowance of birth companions would minimizing the burden of care on healthcare providers and possibly reduce health professional burnout which may precipitate disrespectful care. It also emerged that not being allowed birth companions was considered by adolescent parturients to be one of more severe forms of mistreatment by providers (45). Unanimously, the bodies of work highlighted health professionals' acknowledgement of the need for specialized care in adolescent healthcare delivery; recognizing that adolescents are an especially vulnerable sub-population of parturients with unique needs. All the provider perspectives reiterated a need for care that is personalized, non-judgmental and culturally acceptable to adolescents. Across studies, health professionals requested for interventional training to develop special behavioral skills in providing respectful maternity care for adolescent mothers as a unique sub-group of clients.

### RMC interventions

Studies which offered insight on the design, implementation and evaluation of RMC interventions were generally aligned around three main strategies: shifts in policy and practice to compel improvement health provider behaviors (31), infrastructural improvements in the health system (35, 37) and involvement of the facility-user community (48). Studies that implemented adolescent centered interventions reported increased levels of satisfaction with care (49, 50). One of the studies utilized a multi-faceted intervention with the formation and specialized training of a multidisciplinary “champion team” on adolescent-centered care, as well as policy, attitudinal and infrastructural changes to facilities to make them more adolescent-friendly, especially with regards to confidentiality (49). The study's inclusion of facilities within the same geographical area may have yielded some form of contamination, the level of which could not be ascertained by the study. The study also recorded some decline in participation over time which was attributed to waning engagement or survey fatigue or other indeterminate reasons. This interesting correlate of time is an important lesson to consider in the future design of interventions

Another intervention entailed the implementation of a quality improvement change package intervention which included behavioral trainings and BCC materials for staff aimed at improving hygiene, privacy, and provider-client rapport (50). The authors described a “halo-effect” from the intervention in that the targeted improvements of the intervention led to satisfaction with other non-targeted aspects of care. The study also found that the model is a cost-effective means of improving the quality of maternal care. The study was limited in its inclusion of a small number of facilities and possible contamination from pre-existing quality of care initiatives. The study also cited a possible Hawthorne effect on the facility staff who may have modified their behavior once they knew they were being assessed.

## Discussion

This systematic review aggregated and synthesized literature on respectful maternity care concepts, policies and interventions with a focus on adolescents. The concept of RMC from both adolescents and health provider perspectives were included. The results and recommendations from studies that implemented RMC interventions were also collated and compared. Due to a paucity of quantitative information on RMC incidence and other related factors, this review was unable to include a meta-analysis. This revealed an important gap in information on the quantified success of RMC interventions. However, the included qualitative results were very revealing and helpful in conceptualizing the need for RMC in adolescent obstetric care. In the systematic review, studies universally demonstrated synchrony between adolescent and provider perspectives and knowledge about what RMC entailed. Both groups of studies with adolescents and or health provider participants presented results that described RMC in terms that centered adolescents' comfort, respect and emotional wellbeing in addition to desirable clinical outcomes. Consistently, adolescents across all studies associated a better quality of care with emotional and or psychosocial support. Furthermore, both broad groups of study participants agreed that health professionals typically were untrained in providing the kind of desired interpersonal care necessary to give adolescents a full quality birth experience. This underscores a necessity for interventions which equip professionals with information and behavioral skills that meet the special needs of adolescents. Among the suggested priority areas for intervention in the included studies were birth companionship and adolescent-friendly relationships.

Research evidence has successfully reported improvements in RMC delivery with attendant beneficial outcomes to mothers and their neonates. This has been achieved employing a diverse range of interventions and implementation strategies. A program in Kenya recorded improvements in respectful maternity care provision after implementing interventions that teach and encourage women to understand, own and assert their sexual reproduction health rights (51, 52). Other studies have also employed the strategy of encouraging and facilitating women to seek legal recourse against the perpetrators of intrapartum mistreatment (19). Yet other studies have also employed community involvement as an effective means of mitigating mistreatment by educating and engaging community members to be custodians of the local healthcare system, and to effectively advocate RMC for women in the communities who utilize the health facilities (52).

Despite studies reporting the general success of RMC interventions in mitigating intrapartum mistreatment and consequently, improvements in the quality of maternal care, relatively little evidence exists about interventions that work, (or do not) specifically among adolescent populations who may not necessarily be able to benefit in the same way their older counterparts do. For instance, whilst adolescents generally demonstrate an appreciable awareness of their rights to quality SRH, they may be unable to fully exercise these rights due to certain factors such as younger age and societal biases against engagement in early sex (26). Another prime example is the inability of adolescents, who are often unemployed and socio-economically poorer, to typically afford the legal fees involved in prosecuting a perpetrator of mistreatment. Several more of these barriers exist and evidently create a health inequity for parturient adolescents. In order to effectively mitigate these gaps in serving adolescent mothers, there is a need to analyse and learn from the available evidence, the most effective design and implementation strategies in promoting RMC, specifically to meet the needs of adolescents. This is particularly important as adolescents bear a disproportionately elevated risk of maternal mortality and morbidity (43, 45), and it is paramount that the highest level of quality peripartum care be provided to them to encourage facility-based births and by extension improve their clinical and psychosocial maternal and neonatal outcomes.

## Conclusion

Despite a dearth of evidence on research which seeks to improve maternal care, the review found that very few focus on the aspect of respectful maternity care as an element of quality of care. Moreso, the review highlights an even greater gap in existing adolescent-focused interventions. These interventions have mainly involved policy and programmatic efforts to inspire behavioral change of providers, health system infrastructural improvements and some level of involvement of the facility-user community. Results of these interventions have been positive with recorded improvements and satisfaction in the quality of care. They have also been found to be cost-effective improvements to the health system, with non-targeted improvements being recorded in the quality of maternal care. However, findings from this review indicate that these interventions have been found to be time-dependent, with diminishing returns over time, which gives some insight into future programmatic design. The challenges of these interventions also being affected by pre-existing or concurrent interventions is noteworthy for future design, implementation and evaluation of programmes and policies. This review firstly contributes to efforts to mitigate maternal mortality and morbidity, especially among adolescents who are a key risk group. The review also provides insight into the interventions that prioritize adolescents, the challenges present in their implementation, and the strategies that have facilitated their successful outcomes. This review further offers evidence-based recommendations for the future development of adolescent targeted health policy, research and programmes. A limitation of this review is the inability to conduct a meta-analyses due to the heterogeneity of the included quantitative studies.

## Data Availability

The original contributions presented in the study are included in the article/**Supplementary Material**, further inquiries can be directed to the corresponding author.
