# Supplementary material for: Are respectful maternity care (RMC) interventions effective in reducing intrapartum mistreatment against adolescents? A systematic review
Source: Front Glob Womens Health. 2023 Mar 1;4:1048441. doi: 10.3389/fgwh.2023.1048441 (PMC10014999; doi:10.3389/fgwh.2023.1048441)
Supplement: Supplementary file 1 [file Table1.docx]

**Supplementary Material S1: Search Terms for databases**

1. Intrapartum Mistreatment
2. Disrespect and Abuse
3. Respectful Maternity Care
4. Compassionate Maternity Care
5. 1 OR 2 OR 3 OR 4
6. Adolescent
7. Teenager
8. “Intrapartum Mistreatment” and Adolescent (MeSH)
9. “Intrapartum Mistreatment” and Teenager (MeSH)
10. “Disrespect and Abuse” and Adolescent (MeSH)
11. “Disrespect and Abuse” and Teenager (MeSH)
12. “Respectful Maternity Care” and Adolescent (MeSH)
13. “Respectful Maternity Care” and Teenager (MeSH)
14. “Compassionate Maternity Care” and Adolescent (MeSH)
15. “Compassionate Maternity Care” and Teenager (MeSH)
16. 8 OR 9
17. 10 OR 11
18. 12 OR 13
19. 14 OR 15
20. 8 OR 9 OR 10 OR 11 OR 12 OR 13 OR 14 OR 15
21. Adolescent pregnancy
22. Teenage pregnancy
23. Adolescent pregnancy abuse
24. Adolescent pregnancy mistreatment
25. Teenage pregnancy mistreatment
26. 24 OR 26
27. 25 or 26
28. Young mothers
29. Young mothers mistreatment
30. Young mothers abuse health facilities
31. Adolescent abuse health facilities
32. 21 OR 34
33. 22 OR 34
