# Supplementary material for: Are respectful maternity care (RMC) interventions effective in reducing intrapartum mistreatment against adolescents? A systematic review
Source: Front Glob Womens Health. 2023 Mar 1;4:1048441. doi: 10.3389/fgwh.2023.1048441 (PMC10014999; doi:10.3389/fgwh.2023.1048441)
Supplement: Supplementary file 2 [file Table2.docx]

| **Title** | **Author** | **Country** | **Aim** | **Design** | **Sample size** | **Main Findings** | **Conclusions & Recommendations** |
| --- | --- | --- | --- | --- | --- | --- | --- |
|  |  |  |  |  |  |  |  |
|  |  |  |  |  |  |  |  |
|  |  |  |  |  |  |  |  |
|  |  |  |  |  |  |  |  |
|  |  |  |  |  |  |  |  |
|  |  |  |  |  |  |  |  |

**Supplementary Material S2: Data Extraction Tool**
