# Supplementary material for: Are respectful maternity care (RMC) interventions effective in reducing intrapartum mistreatment against adolescents? A systematic review
Source: Front Glob Womens Health. 2023 Mar 1;4:1048441. doi: 10.3389/fgwh.2023.1048441 (PMC10014999; doi:10.3389/fgwh.2023.1048441)
Supplement: Supplementary file 3 [file Table3.docx]

**Supplementary Material S3: Results of data extraction**

| **Author, Country** | **Aim** | **Design** | **Sample size** | **Main Findings** | **Conclusions & Recommendations** |
| --- | --- | --- | --- | --- | --- |
| Decker et al (2021)  USA & Mexico, | To compare maternity care experiences of Mexican-origin adolescents from both youth and healthcare provider perspectives. | Qualitative individual & group interviews | 74 pregnant / parenting adolescents + 15 providers | Four themes emerged regarding patient-provider interactions: the need for communication and clear explanations, respectful versus judgmental providers, engaging youth in decision making, and a focus on the age of the youth and their partners. Perspectives varied between youth and providers, with providers in both locations identifying several structural challenges in providing quality care to adolescents. | There are several structural and systemic challenges that HCW face in providing quality care to adolescents. There is a need for interventions to address these barriers |
| Duggan & Adejumo (2012) South Africa | To explore adolescent maternity clients perceptions of maternity care and to identify important characteristics of an adolescent-friendly maternity service. | Qualitative individual & group interviews | 18 adolescent maternity clients | Findings fell into 3 categories: AMC-HCP interaction; health care system; and health education. AMCs wanted HCPs to be caring and supportive, use appropriate interaction & body language to make adolescents feel respected and comfortable. Adolescents expected shorter clinic waiting times & more comfortable waiting rooms & stressed the importance of having extra support during labour and birth. | The relationship between the HCP & AMC is essential to ensuring an optimal outcome for mother and baby. Careful consideration needs to be given to how the health care facility and system are set up to ensure comfort of the AMC |
| Dynes et al (2015) Tanzania | To quantitatively identify patient and delivery factors associated with RMC and model the influence of provider characteristics on RMC. | Cross-sectional surveys | 249 providers + 935 post-delivery clients. | Significant client-level determinants for perceived Friendliness/Comfort/Attention RMC included age and self-reported complications. Significant provider-level determinants included perception of fair pay, cadre and number of deliveries in the last month. | These findings illustrate the value of including both client and provider information in the analysis of RMC. Strategies that address provider-level determinants of RMC (such as equitable pay, work environment, access to mentoring platforms) may improve RMC and subsequently address uptake of facility delivery |
| Fuzy et al (2020) South Africa | Explore lived childbirth experiences of middle adolescent age in the western cape province of SA | Qualitative individual interviews | 6 adolescents | Preservation of personhood, unpreparedness for childbirth & unsettled mindset during childbirth are most important aspects of childbirth for adolescents | Healthcare workers must be trained to deliver adolescent-friendly services |
| Gentry et al (2010) USA | To explore the services doulas, provide for disadvantaged pregnant and parenting adolescents | Mixed methods | 30 Doulas | Doulas recognize unequal treatment pregnant and parenting adolescents experience in both public and private spaces. This disparity prompts doulas to take on additional roles to fill social, emotional, and economic voids in the adolescents’ lives. Additional role- taking among doulas can be divided into three categories of role types: (1) family and friend, (2) social and health service provider and advocate, and (3) general life coach and counsellor | Birth support doulas give adolescent mothers valuable assistance by taking on various roles beyond traditional duties associated with being a doula. |
| Hackett et al (2019) Ghana & Tanzania | Increase understanding of adolescents’ experiences with, and perceptions of, ANC and explore how these perspectives might be applied towards future initiatives to enhance adolescent care-seeking behaviour | Qualitative group interviews | 112 | Health service delivery factors influencing ANC uptake among adolescents include i. Health system policies, ii. Patient-provider relationships, iii. Human & material sources iv. Health Centre Practices | Positive interactions with ANC providers may be the most important determinant of perceived quality of care. To ensure that ANC is accessible to adolescents, it is essential to address their particular needs and preferences. |
| Harrison ME, Clarkin C, Rohde K, Worth K, Fleming N. | To share pregnant and parenting youth’s experiences with health care in order to inform recommendations for promoting youth-friendly medical encounters. | Qualitative group interviews | 26 | Three major themes emerged regarding adolescents experiences with health care providers: characteristics of negative health care encounters, the emergence of contemporary stereotypes during these encounters, and characteristics of positive health care encounters. Negative encounters often resulted from perceived judgmental attitudes of providers and were shown to contribute to a general sense of mistrust and fear. Positive health care encounters tended to feature mutual respect, support, open dialogue, and non-judgmental attitudes | In order to engage this high-risk population in health care, practitioners are encouraged to consider their own biases when servicing this population and work towards fostering positive, non-judgmental interactions and supportive environments |
| Jonas et al (2016) South Africa | Gain an understanding of nurses’ and midwives’ intentions to provide maternal and child healthcare & family planning services to adolescents in South Africa. | Cross-sectional surveys | 190 | Self-efficacy has a strong positive association with intentions to provide both MCH and FP services, while there is a moderate association with attitude and norms. | There is a need to improve and strengthen nurses’ and midwives’ self-efficacy in conducting both MCH and FP services in order to improve the quality and utilization of the services by adolescents in South Africa. |
| Jonas et al (2019) South Africa | Explore nurses’ & adolescent girls’ perceptions of barriers and needs to access and utilizing sexual and reproductive healthcare services. | Qualitative group interviews | 24 | SRH nurses are generally supportive of adolescents who ask for and use contraceptives. Non-compliance to FP regimens and repeated requests for termination of pregnancies were perceived by nurses as irresponsible behaviours which are particularly frustrating to them and not in concordance with their personal values. The subsequent nurse-adolescent interactions sometimes appeared to hinder access to and utilization of SRH services by adolescents | Nurses perceive certain behaviours of adolescent girls as irresponsible and warrant their negative attitudes and reactions toward them. The negative attitudes and reactions of nurses potentially further compromises access to and utilization of SRH services by adolescent girls in South Africa and requires urgent attention. Adolescent-friendly clinic hours together with youth-friendly nurses is likely to encourage adolescent girls |
| Kanengoni et al (2019) Zimbabwe | Explore women’s experiences and perceptions of disrespect and abuse from maternity care providers in a low resource rural setting in Zimbabwe. | Qualitative individual & group interviews | 20 | Multifaceted and interconnected factors contribute to midwives’ attitudes and behaviours towards their clients. Midwives’ subjective perceptions, women’s social status, and health system constraints in rural and poorly resourced community, often result in inappropriate services, negative attitudes, abusive treatment, and disrespectful behaviour towards women. Poor treatment in maternity care directly contribute to adverse health outcomes and women’s satisfaction with services. | Improving women’s experience of maternal care requires targeted interventions at the interpersonal level between a woman and her health care providers, as well as at the level of the health care facility and the health system |
| Kiti et al (2022) Kenya | Assess whether having continuous support during labour is associated with better person-centred maternity care (PCMC) among women in rural Kenya | Cross-sectional surveys | 865 | Approximately 68% of the women had continuous support. Women with higher education, higher wealth, and those who work for pay were more likely to have continuous support than women with lower education, lower wealth, and without salaried jobs respectively. Continuous support was also more common among women with lower parity, those who delivered in a health centre, and those assisted by a female provider, or a non-skilled attendant compared respectively to their reference groups. Women who are married, educated, wealthier and hold salaried jobs on average had higher PCMC than women who are unmarried, less educated, poorer and without salaried jobs respectively | Given that many health systems in low resource settings are unable to provide continuous support during labour because of privacy concerns, structural interventions are necessary to ensure labour wards are conveniently arranged to accommodate birth companions. Appropriate interventions to sensitize women, family and providers about the role of continuous support during labour are also needed. Targeted PCMC interventions in public health facilities are necessary to ensure equity in the quality of care delivered. This would help increase utilization of maternal health services and lower maternal and neonatal morbidity and mortality |
| Klima (2003) USA | Centring Pregnancy is a model of group prenatal care that provides the assessment, education, and support of pregnant women and may be particularly useful in adolescent populations. The model is described, and the ways adolescents may benefit from Centring Pregnancy’s unique design is discussed | Commentary |  | Centring Pregnancy is a group model particularly suited for adolescents because it combines all the aspects of prenatal care into one comprehensive program, allows for the addition of support providers such as social workers, and provides opportunities for self-care activities and interaction with peers. | Centring Pregnancy is a model of care that values women’s contributions, creates an environment that fosters empowerment, and builds community for women as they become mothers. |
| Lusambili et al (2020) Kenya | Examine women’s experience of disrespectful care during pregnancy, labour, and delivery to promote improved understanding of the actual care conditions & to inform the development of interventions that can lift the standard of care, increase maternity facility use, and improve health outcomes for both women and newborns. | Qualitative individual & group interviews | 40 | Nursing & medical care during labour & delivery were at times disrespectful, humiliating, uncompassionate, neglectful, or abusive. Male health workers were preferred as they were perceived as more friendly and sensitive. Adolescent females were more likely to report abuse during maternity care while women with disabled children reported being stigmatized. Structural barriers related to transportation and available resources at facilities associated with disrespectful care were identified | A focus on quality and compassionate care as well as more facility resources will lead to increased, successful, and sustainable use of facility care. Interpreting these results within a systems perspective, Kenya needs to implement, enforce, and monitor quality of care guide- lines for pregnancy and delivery including respectful maternity care of pregnant women. To ensure these procedures are enforced, measurable benchmarks for maternity care need to be established, and hospitals need to be regularly monitored to ensure these benchmarks are achieved. |
| Mangeli et al, (2018) Iran | Explore the experiences of Iranian adolescent mothers about the maternal role. | Qualitative individual interviews | 18 | Two main strategies including optimizing the process of the maternal experience & use of supporters for the acceptance of the maternal role were identified among adolescents | Early motherhood is an unpleasant experience; if appropriate strategies and facilitators are used, it can have positive consequences. Health care providers should provide satisfactory services in care, education and support with adequate knowledge of adolescent characteristics. Opportunities in the community should also be directed to support this vulnerable group |
| McLeish & Redshaw (2019) England | To explore the maternity care experiences of mothers with multiple disadvantages. | Qualitative individual interviews | 40 | Mothers brought feelings of powerlessness and low self-esteem to their encounters with maternity professionals, which could be significantly worsened by disrespectful care. They needed support to navigate the complex maternity system. Positive experiences were much more likely where the mother had received continuity of care from a specialist midwife or small team | Mothers with multiple disadvantages value being treated as an individual, making informed choices, and feeling safe, but may lack the confidence to ask questions or challenge disrespectful treatment. Training and supervision should enable maternity professionals to understand how confusing maternity care can be to very disadvantaged mothers. It should emphasise the need to provide accessible and empowering information and guidance to enable all mothers to make choices and understand the system. Leaders of maternity services must do more to challenge negative providers attitudes and ensure that that all mothers are treated |
| November & Sandall (2018) Sierra Leone | Explore the causes of high incidence of maternal death for younger teenagers, and to identify possible interventions to improve outcomes. | Qualitative individual & group interviews | 71 | Several obstetric risks were discussed by midwives, but were explicitly related to socio-economic factors. A cross-cutting theme was gendered social norms for sexual behaviour for both boys and girls, being reinforced by significant adults such as parents and teachers. | Findings challenge the notion that adolescent girls have the necessary agency to make straightforward choices about their sexual behaviour and contraceptive use. For girls who do become pregnant, risks are believed to be related more to stigma and abandonment than to physical maturity, leading to lack of family-based support and delayed care-seeking for antenatal and delivery care. Two potential interventions identified within the research are a mentoring scheme for the most vulnerable pregnant girls and a locally managed blood donation register. |
| Bwalya et al (2018) Zambia | Explore and describe the lived experiences of ANC among pregnant adolescents at Kanyama and Matero Referral Clinics in Lusaka, Zambia. | Qualitative individual interviews | 12 | Adolescents experienced both positive and negative ANC. Additionally, other issues reported by adolescents were the unfavourable health facility opening hours, the lack of specific spaces for adolescents, as well as inadequate privacy and confidentiality. Some solutions suggested to overcome some problems include reducing waiting hours/ consultation time at the clinic and allocating specific rooms or spaces for pregnant adolescents | Appropriate interventions targeting pregnant adolescents with emphasis on making ANC more adolescent friendly may improve the quality of and accessibility of antenatal services. The adolescent friendly ANC should integrate health promotion activities aimed at sensitising elderly HCW on the importance of supporting pregnant adolescents |
| Oosthuizen et al (2017) South Africa | Determine women’s experiences of childbirth with a view to improving respectful clinical care practices in low-risk, midwife-led obstetric units | Cross-sectional surveys | 653 | Age, language, educational level and length of residence in the district were significantly associated with disrespectful care. Overall, the following groups of mothers reported more negative care experiences during labour: women between the ages of 17 and 24 years; women with limited formal education; and women from another province or a neighbouring country. With regard to respectful care, 54% of mothers indicated that all providers members had spoken courteously to them, 48% said they had been treated with a lot of respect, and 55% were completely satisfied with their treatment. | There is a need to improve respectful care through interventions that are integrated into routine care practices in labour wards. To stop the spiral of abusive obstetric care, the care provided should be culturally sensitive and should address equity for the most vulnerable and underserved groups. All levels of the health care system should employ respectful obstetric care practices, matched with support for midwives and improved clinical governance in maternity facilities |
| Peters et al (2019) Netherlands | Gaining a deeper understanding of their experiences and satisfaction with antenatal, birthing and maternity care will help to adjust healthcare responsiveness to meet their needs during pregnancy, childbirth and the postpartum period | Qualitative FGD | 106 | The obstetric healthcare systems’ responsiveness in all phases of care (antenatal, birthing and maternity) did not meet these women’s needs. The ‘respect for persons’ domains ‘autonomy’, ‘communication’ and ‘dignity’ and the ‘client orientation’ domain ‘prompt attention’ were judged most negatively. | Conclusions The study findings give contextual meaning and starting points for improvement of responsiveness in the provision of obstetric care within a multi-ethnic women’s population. |
| Peterson et al (2012) Canada | Determine whether hospital-based perinatal nurses with expertise in adolescent mother-friendly care identify a need to improve inpatient nursing care of adolescent mothers and how well perinatal units support nurses’ capacity to provide adolescent mother-friendly care. | Qualitative group interviews | 34 | Key informants rated their own skill in caring for adolescent mothers higher than other nurses. They attributed their expertise working with adolescent mothers to their clinical and life experiences and their ability to develop rapport with adolescents. A common reason for the assigned lower peer-group ratings was the judgmental manner in which some nurses care for adolescent mothers. Key informants also identified that hospital-based perinatal nurses lack adequate knowledge of community-based resources for adolescent mothers, educational programs related to adolescent mother-friendly care were insufficient, and policies to inform the nursing care of adolescent mothers were not available or known to them. | Minority of perinatal nurses have expertise in adolescent mother-friendly care. There is a need for perinatal unit-level interventions to support the development of nurses’ skills in caring for adolescent mothers and their knowledge of community-based resources. Peer mentoring and self-reflective practice are promising strategies. |
| Quosdorf et al (2020) Canada | Explore adolescent-friendly care from the perspective of hospital-based perinatal nurses. | Qualitative group interviews | 27 | Nurses described two main goals: (a) delivering a positive experience and (b) ensuring mother and infant safety. They accomplished these goals by being nonjudgmental, forming a connection, and individualizing nursing care. | This research contributes to our understanding of how hospital-based perinatal nurses engage and support adolescent mothers |
| Reibel et al (2015) Australia | Understand young Aboriginal women’s views on pregnancy care to assist maternity services develop localised pathways that encourage engagement with pregnancy care | Qualitative individual & group interviews | 28 | Typical actions indicative of antenatal engagement included: female relatives directing young woman to care; availability at Aboriginal Health Service/ public hospitals and community-based settings of multidisciplinary teams and, a continuous relationship with known and trusted care providers. Factors such as relocation for childbirth may interrupt pregnancy care. Active measures such as providing appointment reminders and transport to and from appointments assists young women to maintain antenatal contact | The role of female relatives in directing young women’s engagement with pregnancy care is crucial combined with availability of known and trusted care providers. Relocation from a home community to the nearest birth facility, and associated accommodation and transport options, are causes of concern requiring health system changes which more fully support culturally safe maternity options regardless of location |
| Riley et al (2018) USA | Evaluate the effects of implementing the Adolescent Champion model, a novel quality improvement program targeted at helping primary care sites become more adolescent-centered | Quasi-experimental | 474 | Adolescent patients’ experiences with both provider and site overall significantly improved. Providers perceived an improvement in clinic practices relating to adolescents and in their ability to make institutional and personal change. The majority of changes were sustained 1-year postintervention. | Implementing the Adolescent Champion model successfully helped primary care sites become more adolescent-centered. Further studies are needed to evaluate the effects of this model on patient outcomes. |
| Sudhinaraset et al (2021) India | Describe the impact of spreading a Change Package, or interventions that other health facilities had previously piloted and identified as successful, to improve PCMC in public health facilities | Quasi-experimental | 1200 | Out of a 100-point scale, a 24.93 point improvement was observed in overall PCMC scores among spread facilities compared to control facilities from baseline to endline. For the eight PCMC indicators that the Change Package targeted, spread facilities increased 33.86 points relative to control facilities across survey rounds. | Findings suggest that spread of a PCMC Change Package results in improved experiences of care for women as well as secondary outcomes, including clinical quality, nurse and doctor visits, and decreases in delivery problems |
| Wilson-Mitchell et al (2018) Jamaica | Explore the perceptions of midwives who provide care for adolescent mothers in Jamaica. | Qualitative individual & group interviews | 12 | Participants shared their work experience with adolescent mothers and explained that restrictive public and institutional policies, culture, personal beliefs, and the location of care delivery hindered them from providing respectful care which allows for shared decision- making, informed consent, and allowing for a desired labour companion. | Out of hospital environments appeared to facilitate the advocacy role. Healthcare policies and provider attitudes had the potential to hinder or to promote RMC. |
| Dynes et al (2015) Tanzania | Collection and analysis of interviews linked between clients and providers for description of patient and provider characteristics and their association with RMC | Cross-sectional surveys | 1184 | Significant client-level determinants for perceived Friendliness/Comfort/Attention RMC included age, self-reported complications labour companionship and religiosity. Significant provider-level determinants included perception of fair pay, cadre, work hours and number of deliveries in the last month. | These findings illustrate the value of including both client and provider information in the analysis of RMC. Strategies that address provider-level determinants of RMC may improve RMC and subsequently address uptake of facility delivery |
| Heartfelt (2007) Sweden | Describe Swedish midwives’ reflections on their experiences of caring for teenage girls during pregnancy and childbirth | Qualitative group interviews | 24 | Two main themes, with three sub-themes each, were generated by the midwives: (1) the teenage mother, as ‘teenagers who are proud of becoming mothers’, ‘teenagers who are unprepared for becoming mothers’ and ‘teenagers with an immigrant background’, and (2) ‘the midwives’ wish to care for the teenage mother’, as ‘taking the teenage mother seriously’, ‘being an important person for the teenage mother’, and ‘being a help for the teenage mother | The findings provide some understanding of the unique characteristics of caring for teenage mothers. For midwives, the most important aspects of caring for the teenage mother included taking the teenage mother seriously, allowing the midwife herself to become an important person for the teenage mother, and being a help to the mother |
| Jittitaworn et al (2020) Thailand | Understand the experiences of healthcare professionals in caring for pregnant adolescent women in Thailand. | Qualitative individual interviews | 21 | The core concept ‘recognising the challenges of providing care for young Thai pregnant women’ explained the provision of care. This concept contained three main themes: 1) having an awareness of the political and societal contexts and environment of care; 2) being aware of attitudes and the need to develop psychosocial skills in caring for adolescent women; and 3) having different approaches to caring for pregnant adolescents. A lack of continuity of care was a significant barrier in terms of structure and process. Effective communication was important to provide quality care. | Healthcare professionals recognised barriers to providing effective care for adolescent women. These findings may inform healthcare professionals and policymakers in relation to the systems of care required in addressing the needs of pregnant adolescents to meet the goal in providing a positive pregnancy experience for all women |
| Peterson et al (2007). Canada | Describe adolescent mothers’ satisfactory and unsatisfactory inpatient postpartum nursing care experiences | Qualitative individual interviews | 14 | Adolescent mothers’ satisfaction is dependent on their perceptions of the nurse’s ability to place them “at ease.” Nursing care qualities that contributed to satisfactory experiences include nurses’ sharing information about themselves, being calm, demonstrating confidence in mothers, speaking to adolescent and adult mothers in the same way, and anticipating unstated needs. Unsatisfactory experiences hindered development of an effective nurse-client relationship. | These findings illustrate the value of qualitative inquiry for understanding patients’ satisfaction with care & have implications for nursing education programs |
